# Pyorrhœa Alveolaris.—Personal Experence in Treatment

**Published:** 1899-05

**Authors:** W. G. Beers

**Affiliations:** Montreal.


ARTICLE IX.

### PYORRHŒA ALVEOLARIS.—PERSONAL EXPERIENCE IN TREATMENT.

W. G. BEERS, L. D. S., D. S., MONTREAL.

Read before the Maritime Dental Association, Sept. 1898.

What we positively know about the pathology of pyorrhœa alveolaris might cover half a sheet of note paper. What we positively do not know would fill folios. Our investigations are like a deep dive into a dark sea, knowing nothing of the bottom, and uncertain of rescue; and while the regulation "twenty minutes" ought to suffice to tell all I know of my own experience in treatment, it would take hours to relate explorations in the realms of pathological speculation, and the many discoveries made of one's own etiological ignorance. We make a step towards knowledge when we discover and confess our ignorance, and perhaps one's frankness in this direction may be a little inspired by the feeling that his friends are in the same plight. Somebody said that while caries is the most prevalent disease in existence, there are more teeth lost by pyorrhœa alveolaris; but we have no statistical proof that this is true; and I am disposed to accept it as one of those hasty and epigrammatic statements, which pass too current entirely because of their catching phraseology. We cannot assume that all teeth that are "lost" are lost legitimately. There are more legitimate deaths than births. It is quite safe to suggest that thousands of teeth are "lost" by ignorance on the part of the patient or malpractice on the part of the dentist. It needs no extensive experience to assert, that the large proportion of "lost" teeth in the establishment of the quack dentist are like lost souls that have none but themselves to blame for their damnation.

We need no statistical proof to declare that the disease which attack the soft and adjacent structures of the mouth, especially those intimately connected with the alveoli, are more difficult to treat, and more likely to recur, than those which attack the teeth themselves. The force of resistance (*vie medicatrix naturæ*) aid the soft structure; it is entirely absent in the hard, unless in the very exceptional and limited cases of arrested caries. Yet there is a limit to the tolerance which the soft structures exhibit, and when that limit is passed we observe irretrievable recession of gum, alveolar margins, entire destruction of the sockets and pericementum. There are a hundred questions which might be referred to, but however we may speculate on the etiology of this disease, I venture to express my belief, that if we take care of the gingival line, where the enamel and cementum join, or, in brief, of the gum tissue they would have no pyogenic bacteria in their mouths.

The etiology of caries is no longer a speculation; that of pyorrhœa alveolaris is entire so. We know that it is a perversion of normal phpsiological action; that it is a deviation from the normal standard of health of the parts concerned, constituting a distinct disease that may end in the death and loss of the teeth. But we are not agreed as to whether or not it is infectious, local, constitutional, or both. It is a much named disease; whether it be called Riggs' Pyorrhœa, Alveolaris, Suppurative Inflammation, Phegedenic, Pericementitis. Is it due to salivary or sanguinary calculus, or both, or fungi, or bacteria? Is it a local expression of a systemic condition, and located in the mouth because of a predisposition of the gums, the pericementum and the alveoli? Is it a primary or secondary lesion? Is it never present, excepting in a depraved state of the nervous system? Is it associated with gout, rheumatism, Bright's disease, locomotor ataxia, uterine troubles; and other constitutional complications, the excessive use of salt, or alcohol, which causes increased secretion of uric acid? Is the calculus uric acid? Is it connected with catarrh of the mucus membrane of the nose or pharynx, rachitis, tuberculosis, scorbutis, scrofula, chronic constipation, exanthematous diseases, malaria, diabetes, tabes dorsalis, dyspepsia, syphilis, anæmia, chlorosis, repeated pregnancy, bad air, bad food, hygienic neglect? Or will some Boston prophet arise, who will tell us that it and all other oral troubles are due to the presence of amalgam, as in the middle ages, earthquakes, tempests and epidemics were ascribed to the devil or the Jews. With all this bewilderment in etiology, how can our treatment be based upon anything better than empiricism? If the etiology is obscure, how can treatment be scientific? We must just console ourselves by feeling our way in the dark, and taking the consequences until some one lets in the light. Someway of treatment where the disease has all the pathogmonic characteristics; but, to be as brief as possible, let me suggest a few aphorisms. Keep the mouth and your hands and whatsoever you see perfectly aseptic. Whatever drugs you use be sure they are pure. You must needs work a good deal in the dark, but get all the light possible both in diagnosis and in operating. A drug that is useful may be abused by over-use so as to become a positive irritant. Iodine and aconite in the incipient stage of inflammation will retard the circulation and stimulate lymphatic action, but used to excess, as it commonly is, it is a destiuctive poison. Alkalies in any form should not be used, either by the patient or the operator, because they percipitate the salts of common tartars. In secondary treatment; and in fact, all through, be careful not to disturb granulations which you have been trying to produce. Do not operate oftener, as a risk than three days of each week; some rest is as necessary as some operating.

Before beginning the removal of serumal calculus, be as sure as possible just where it lies. There may not be any. I have seen acid conditions in pregnancy, etc., when salivary calculus for the time is dissolved and disappears. As to whether or not this occurs where the calculus is serumal, I do not know. Massage the gum with the finger to make it bleed, if it will; sometimes lance; sometimes use a leech. Relieve venous congestion as much as possible before scaling. There is an object in manipulating the gum margins so as not to wound them any more than they are already wounded. I begin my diagnosis for the nodules of calculus by explorers I have made for the purpose, using a diagram of the roots to mark the parts upon which it is deposited. My pocket hoes and scrapers I have made so as to adopt them as nearly as possible to the contour of the roots. Square hoes and scrapers are not the best. I have had these made in duplicate of platinum-iridium so as to use them when the sulphuric acid is injected into the pockets. Some hydrogen, etc., and as I wish to leave room for somebody else to say something, I will say no more.—*Dominion Dental Journal.*

